# Transmission of airborne virus through sneezed and coughed droplets

**DOI:** 10.1063/5.0022859

**Published:** 2020-09-01

**Authors:** Santosh K. Das, Jan-e Alam, Salvatore Plumari, Vincenzo Greco

**Affiliations:** 1 School of Physical Sciences, Indian Institute of Technology Goa, Ponda 403401, Goa, India; 2 Variable Energy Cyclotron Centre, 1/AF Bidhan Nagar, Kolkata 700064, India and Homi Bhabha National Institute, Training School Complex, Mumbai 400085, India; 3 Department of Physics and Astronomy, University of Catania, Via S. Sofia 64, I-95125 Catania, Italy and Laboratori Nazionali del Sud, INFN-LNS, Via S. Sofia 62, I-95123 Catania, Italy

## Abstract

The spread of COVID19 through droplets ejected by infected individuals during sneezing and coughing has been considered a matter of key concern. Therefore, a quantitative understanding of the propagation of droplets containing the virus assumes immense importance. Here, we investigate the evolution of droplets in space and time under varying external conditions of temperature, humidity, and wind flow by using laws of statistical and fluid mechanics. The effects of drag, diffusion, and gravity on droplets of different sizes and ejection velocities have been considered during their motion in air. In still air, we found that bigger droplets traverse a larger distance, but smaller droplets remain suspended in air for a longer time. Therefore, in still air, the horizontal distance that a healthy individual should maintain from an infected one is based on the bigger droplets, but the time interval to be maintained is based on the smaller droplets. We show that in places with wind flow, the lighter droplets travel a larger distance and remain suspended in air for a longer time. Therefore, we conclude that both temporal and geometric distance that a healthy individual should maintain from an infected one is based on the smaller droplets under flowing air, which makes the use of a mask mandatory to prevent the virus. Maintenance of only stationary separation between healthy and infected individuals is not substantiated. The quantitative results obtained here will be useful to devise strategies for preventing the spread of other types of droplets containing microorganisms.

## I. INTRODUCTION

It is common knowledge that droplets released through coughing, sneezing, speaking, or breathing contain microorganisms (bacteria, virus, fungi, etc.) causing a large number of diseases.^1^ The droplets containing pathogens^2^ can transmit from an infected individual to a healthy one in several ways,^3^ such as through the respiratory system in the form of droplets or aerosols or via direct contact (touching a contaminated hand rail, a hand shake, etc.). The determination of the abundance of viruses in air,^4^ their effectiveness to infect,^5^ their survivability on the surface of different types of materials,^6^ and contrasting among the routes of transmission remain a big challenge; therefore, these factors limit our ability to evaluate the risk.^7^ Apart from coughing and sneezing, the release of viruses through respiration^8,9^ and speaking is well known.^10,11^ Interestingly, it has been pointed out in Ref. 10 that a large number of droplets carrying pathogens can be emitted through human speaking, and the emission intensifies with the loudness of speech and such a mechanism of emission, though independent of the language spoken, depends on some unknown physiological factors varying among individuals. The statistical mechanics and fluid dynamics play crucial roles in understanding the propagation of the droplets. Fluid dynamical tools have been applied to understand the aeorsolization and propagation of human droplets.^12,13^ The techniques of the stochastic statistical mechanics become particularly useful for the study of the motion of aerosols (droplets with a diameter of 5 *μ*m^14^) for which the airborne transmission turns out to be very vital. The aerosols undergo random Brownian or diffusive motion in air, which can be studied within the scope of the Langevin differential equation as it contains a stochastic source term, which is normally ignored in the Eulerian–Lagrangian approach.^12^

In the present work, we investigate the space–time evolution of these droplets by taking into account the diffusive force through the Langevin equation. The diffusive force plays a crucial role, particularly in the motion of small droplets in air. This will help enormously in planning the preventive strategies of the virus carried by the droplets. The motion of the droplet ejected in air with some initial velocity at some spatial point will interact with the molecules of air. The problem will not only be complex but unsolvable if one considers the interaction of the droplet with the individual molecules of the air, which are changing positions continuously, resulting in continuous change in the interacting force. In such a situation, the air molecules can be regarded as forming a thermal bath characterized by temperature and density where the droplets are in motion. The interaction of the droplets with the bath can then be lumped into an effective force, which contains drag and diffusive terms. Therefore, the interaction of the droplets with air can be taken into account through the drag and diffusion coefficients. The facts stated above set an appropriate stage to study the propagation of sneezed and coughed droplets in air within the scope of the Langevin stochastic differential equation of statistical mechanics.^15,16^ It is crucial to note that the Langevin equation can be applied to solve the problem under study because the mass of the droplets is much higher than the mass of the oxygen and nitrogen molecules present in air. After the ejection, the change in the position of the droplets with time will be governed by the (1) drag force exerted by air on the droplet, (2) diffusive force, and (3) gravitational force acting on them. The inclusion of all these forces enables us to study the trajectories of droplets in a wide range of sizes. The climatic conditions affect the transmission of droplets in air (see Ref. 17 for details). The thermophysical properties of air vary from place to place depending on the temperature and relative humidity. These variations have been taken into consideration through the temperature^18^ and relative humidity^19^ dependence of the viscosity of air. The viscosity of air has been used to estimate the drag coefficient by employing the Stokes formula.^20^ The Einstein fluctuation–dissipation relation^16^ has been used to calculate the diffusion coefficient, which is directly proportional to the temperature. Therefore, the temperature and humidity dependence of the space–time evolution of the droplets enter the calculation through the drag and diffusive forces exerted by air on the droplets.

The trajectories of the droplets will be different in still and flowing air [such as in an air conditioned (AC) room]. The propagation of the droplets in a quiet indoor setting^21^ is very different from a room with AC ventilation. The direction of air flow due to AC ventilation plays a vital role.^22,23^ The present study considers both the cases—situations with still air and wind flow. The flow of air has been taken into account by using the Galilean transformation of the Langevin equation. The velocity of the droplets will dissipate in air in the course of time. It is expected that the gravitational force is superior to both the drag and diffusive forces for large (massive) droplets. However, for smaller droplets, drag and diffusive forces will predominate. Therefore, it is interesting to study how these competitive forces influence the distance that the droplets traverse from the source (infected individual) and for how long they remain suspended in air. This will indicate the distance (both geometric and temporal) that a healthy individual should maintain from an infected individual to prevent virally transmitted diseases.

## II. METHODS—SOLVING THE LANGEVIN EQUATION NUMERICALLY BY MONTE CARLO TECHNIQUE

We write down the following Langevin equations for the motion of the droplets of mass (*M*) in the still air in the presence of gravitational field:^15^

$$\frac{dr_{i}}{dt}=v_{i},$$ (1)

$$M\frac{dv_{i}}{dt}=-\lambda v_{i}+\xi \left(t\right)+F^{G},$$ (2)

where *dr*_*i*_ and *dv*_*i*_ are the shifts of the coordinate and velocity in each discrete time step *dt*, respectively, and *i* stands for the Cartesian components of the position and velocity vectors. The *λ* in Eq. (2) is the drag coefficient. The first term in the right hand side of Eq. (2) represents the dissipative force, and the second term stands for the diffusive (stochastic) force where *ξ*(*t*) is regulated by the diffusion coefficient *D*. *ξ*(*t*) is also called noise due to its stochastic nature. We study the evolution with a white noise ansatz for *ξ*(*t*), i.e., ⟨*ξ*(*t*)⟩ = 0 and ⟨*ξ*(*t*)*ξ*(*t*′)⟩ = *Dδ*(*t* − *t*′). White noise describes a fluctuating field without memory, whose correlations have an instantaneous decay, called *δ* correlation. The third term in Eq. (2), *F*^*G*^, represents the gravitational force (=*Mg*, *g* = 9.8 m/s^2^) acting on a droplet of mass *M*.

The Galilean transformation has been used to take care of the flow of air [with velocity *u*(*x*)] into the Langevin equation. In the present work, our aim is to study how the dynamics of droplets are affected by the flow of air. Therefore, we conceive a velocity profile for air flow as $u\left(x\right)=u_{0}\left(1-\frac{x}{x_{\text{max}}}\right)$ to serve this purpose, where *x* is the running coordinate, *u*_0_ is the peak value of *u*(*x*) at *x* = 0, and *x*_max_ is the maximum value of *x*, which may be constrained by the size of an AC room. However, a more complex velocity profile can also be contemplated. We have taken the flow velocity along the horizontal direction with vanishing components along upward and downward directions. It is obvious that any non-zero upward (downward) component will enhance (reduce) the time of suspension of droplets in air.

We solve the Langevin equations [Eqs. (1) and (2)] simultaneously by using Monte Carlo techniques^24,25^ with the inputs discussed below. For the initial spatial coordinate we use, *x* = *y* = 0 and *z* = *H*_0_, where *H*_0_ is the height (taken as 1.7 m) at which the droplet is released (nose/mouth), that is, the initial spatial coordinate of the droplet is (*x*, *y*, *z*) = (0 m, 0 m, 1.7 m). We distribute the initial velocity uniformly in the *x*–*y* plane, where *v*_*z*_ = 0. The gravitational force acts on the downward *z* direction. We vary the radius (*R*) of the droplets from 2.5 *μ*m to 100 *μ*m^26^ and the ejection velocity (*V*_0_) from 5 m/s to 21 m/s.^27^ The mass of the droplet has been estimated from the radius (*R*) by using the relation *M* = 4*πR*^3^*d*/3, where *d*(=997 kg/m^3^) is the density of the droplet. The value of the drag coefficients, *λ*, is estimated by using the relation, *λ* = 6*πηR*, obtained from the Stokes formula. The value of the diffusion coefficient is obtained by using the Einstein relation,^16^ *D* = *K*_*B*_*Tλ*, where *K*_*B*_ = 1.38 × 10^−23^ J/K is the Boltzmann constant and *T* is the temperature. We consider $L\left(t\right)=\sqrt{x{\left(t\right)}^{2}+y{\left(t\right)}^{2}}$ as the horizontal distance traveled by the droplet from the point of ejection and the maximum value of *L*(=*L*_max_) dictates the stationary distance to be maintained between infected and healthy persons to avoid the spread of virus.

It may be mentioned that if we set the values of drag and diffusion coefficients to zero, then our numerical results are in excellent agreement with the results obtained by assuming free fall of the droplets with a large size (mass).

## III. RESULTS

**FIG. 1.**
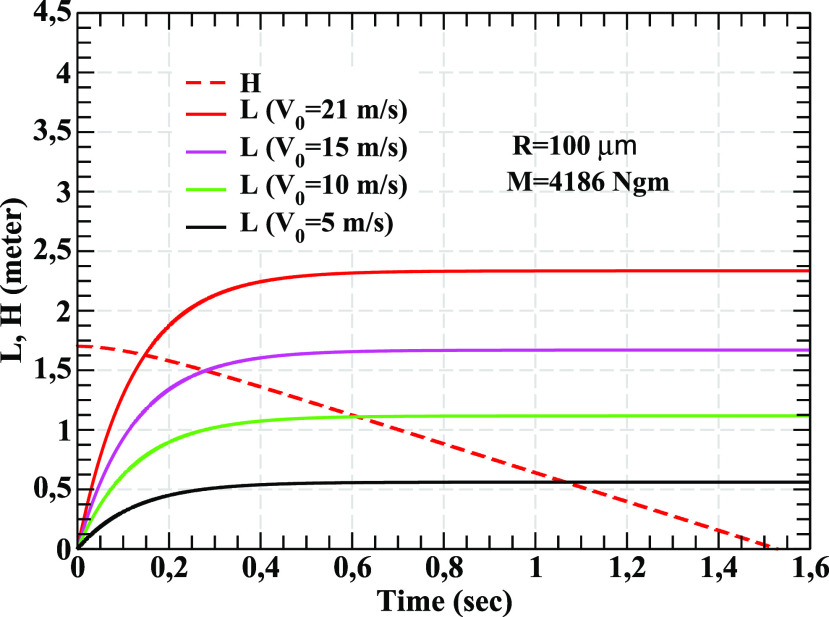
The horizontal distance, *L*(*t*), traveled by the ejecta of mass 4186 ng (nano-g) from the source of infection as a function of time for different ejection velocities has been shown here. The droplets are ejected at a height of 1.7 m from the ground. The change in the height, *H*(*t*), with time of the droplet for initial velocity 21 m/s has also been depicted.

**FIG. 2.**
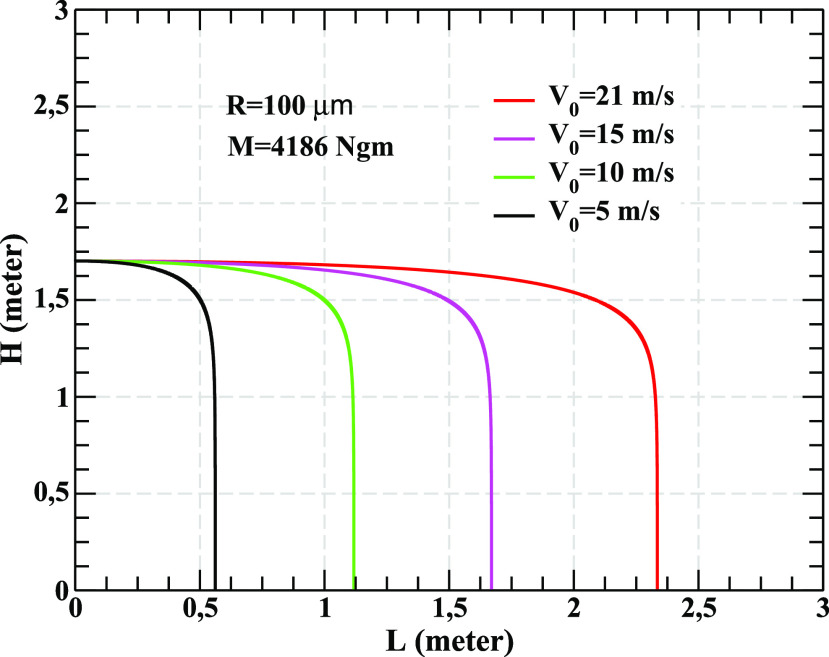
Variation in *H* with *L* for a droplet of 4186 ng mass and 100 *μ*m radius for different ejection velocities.

**FIG. 3.**
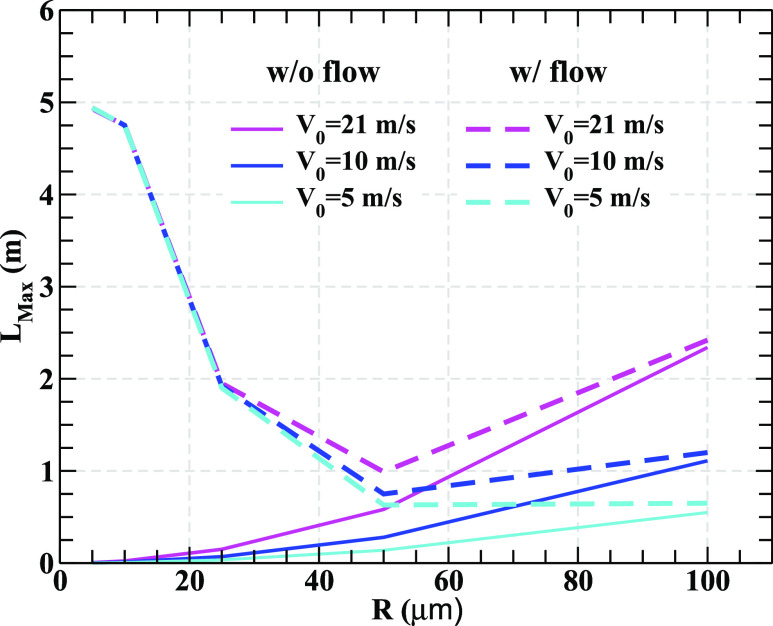
The variation in the maximum horizontal distance (*L*_max_) traveled by droplets as a function of radius for different ejection velocities.

Among other factors, the contamination depends on the mass and initial velocity of the droplets. However, the droplets ejected through coughing and sneezing will have different sizes (and, hence, masses) and initial velocities. Therefore, we provide results for a range of droplet sizes and initial velocities. The contagion by the droplets will also depend on air flow, temperature, and humidity of air where the droplets are discharged. Sensitivities of the results to these factors have also been investigated and discussed below. The results presented in Figs. 1 and 2 have been obtained in still air at temperature, *T* = 30 °C with inputs discussed above. In Fig. 1, the time variation in the horizontal distance (*L*) traveled by droplets at various ejection velocities has been displayed. The (horizontal) distance, *L*, traveled by the droplets from the source depends strongly on the initial velocity and mass. While a droplet of mass 4186 ng with small ejection velocity, *V*_0_ = 5 m/s, travels a distance, *L* ≈ 0.55 m, a droplet with larger velocity, *V*_0_ = 21 m/s, travels 2.35 m approximately. This droplet takes about 1.5 s before it settles on the ground under the action of gravity. Other droplets with intermediate values of *V*_0_ = 15 m/s and 10 m/s travel horizontal distances ∼1.7 m and 1.1 m, respectively. It may be mentioned here that a droplet of radius 200 *μ*m takes about 0.73 s to fall on the ground under the action of gravity, which may be compared with the value for free fall time [$t=\sqrt{\left(2H\right)/g}=0.59\text{ s}$] from a height of 1.7 m (please also see Refs. 28 and 29). This indicates that the free fall under gravity will be a reasonable approximation for droplets having radii larger than 200 *μ*m. The red dashed line in Fig. 1 shows the variation in the height, *H*(*t*), with time when it is released at an initial height, *H*_0_ = 1.7 m at *V*_0_ = 21 m/s. The time variation in *H*(*t*) for large droplets (mass 4186 ng or more) with other values of *V*_0_ are not shown because of its weak *V*_0_ dependence. The results discussed above can be viewed in a different way as follows: Fig. 2 shows the change in the height [*H*(*t*)] of the droplets with horizontal distance [*L*(*t*)] for different initial velocities (*V*_0_). A droplet of mass 4186 ng with *V*_0_ = 21 m/s (5 m/s) travels a horizontal distance, *L* ≈ 2.35 m (0.55 m). The same droplet with intermediate *V*_0_ values, 15 m/s (10 m/s), travels ∼1.7 m (1.1 m). These results indicate that big (massive) droplets falls on the ground within a short time due to the gravitational force but they travel larger distance due to the larger momentum as the drag force for such droplets is weaker than the gravitational force. These results are consistent with the results displayed in Fig. 1. From the preventive strategic point of view, the question to be asked is what is the maximum horizontal distance (*L*_max_) that a healthy individual should maintain from an infected one. The answer will depend on several factors discussed above, e.g., ejection velocity, mass of the droplets, temperature, humidity, and flow velocity of air. We evaluate *L*_max_ both under still and flowing air conditions and display its variation with the radius (*R*) of the droplets for ejection velocities, *V*_0_ = 5 m/s, 10 m/s, 21 m/s in Fig. 3 at a temperature, *T* = 30 °C. It is appropriate to mention here that the mean value of *V*_0_ is about 10 m/s, and the value 21 m/s is close to the highest possible value of *V*_0_ for droplets originating from coughing.^21,27^ The value of *u*_0_ appearing in the velocity profile of the wind mentioned above has been taken as *u*_0_ = 0.1 m/s and *x*_max_ = 5 m.

We observe that the maximum horizontal distance traveled by the droplets in still air increases with its size or mass (Fig. 3). Droplets with larger *V*_0_ give a larger value of *L*_max_ for given *R*. It is crucial to note that *L*_max_ for large (massive) droplets does not change much with moderate air flow. The action of gravity on large droplets dominates over the drag and diffusive forces, and hence, they expeditiously settle gravitationally. In still air, a droplet of 100 *μ*m radius travels 2.35 m, 1.1 m, 0.55 m for *V*_0_ = 21 m/s, 10 m/s, and 5 m/s, respectively. The drag and diffusive forces do not allow small droplets to travel long distance in still air.

However, under the flowing air condition, the scenario is very different. Gravitational force imparts a downward terminal velocity (*v*_*t*_) to the droplet, which is given by *v*_*t*_ = 2*R*^2^(*d* − *ρ*)*g*/(9*η*), where *ρ* is the density of air. In an environment of flowing air with flow velocity *u*(*x*), the resultant of *v*_*t*_ and *u* will dictate how long a droplet will move before gravitationally settled. If *v*_*t*_ of a droplet is large compared to *u*_0_ (peak value of the flow velocity), then it will quickly settle under the action of gravity. The value of *v*_*t*_ for a 100 *μ*m droplet is 1.2 m/s, which is 12 times larger than the peak value of the flow velocity, *u*_0_ (=0.1 m/s); therefore, such droplets will strike the ground fast without much effects of flow. However, the smaller droplets are strongly affected by the flow. The value of *v*_*t*_ for a 5 *μ*m droplet is 0.3 × 10^−2^ m/s, which is more than an order of magnitude lower than *u*_0_(=0.1 m/s). Such small droplets are influenced by drag, diffusion, and flow and have more time to travel large distances.^33^ A droplet of radius 5 *μ*m will traverse a distance of 4.95 m. It is crucial to note that for small droplets, *L*_max_ is insensitive to ejection velocity. A droplet with intermediate size experiences some sort of cancellation between the actions of gravitational and drag force and, therefore, moves a smaller distance if flow velocity is low compared to the corresponding values of their *v*_*t*_ (Fig. 3).

We have also considered *u*_0_ = 0.25 m/s to understand the effect of air flow. We found that the increase in the flow velocity from 0.1 m/s to 0.25 m/s changes the *L*_max_ by 1%, 88%, and 8.2% for droplet of radii 5 *μ*m, 50 *μ*m, and 100 *μ*m, respectively. It is clear that the effect of increase of *u*_0_ on a 5 *μ*m droplet is small because its *v*_*t*_ ≪ 0.1 m/s and, hence, any further increase in *u*_0_ has negligible influence. A 5 *μ*m droplet will travel a distance of 4.95 m (5 m) from the point of ejection if the peak flow velocity is 0.1 m/s (0.25 m/s). Similarly for a 100 *μ*m droplet, the gravitational effect still dominates because their *v*_*t*_ is more than 0.25 m/s, resulting in only about 8.2% increase in *L*_max_. However, a 50 *μ*m droplet has *v*_*t*_ = 0.3 m/s, which is comparable to *u*_0_ = 0.25 m/s, and hence, the change in *L*_max_ for such a droplet is substantial (88%). Therefore, it is important to note that the distance traveled by a droplet will depend on the interplay between the magnitudes of downward terminal velocity and the flow velocity. Therefore, as a preventive strategy, a healthy person should maintain different distances in still and flowing air environments.

We note that for smaller droplets, *L*_max_ ≈ *x*_max_, suggesting that the dynamics of these droplets is almost entirely determined by the air flow. At the distance *L* = *x*_max_, the velocity profile of air turns to zero, and the drag of air becomes dominant, which does not allow the smaller droplets to travel anymore.

**FIG. 4.**
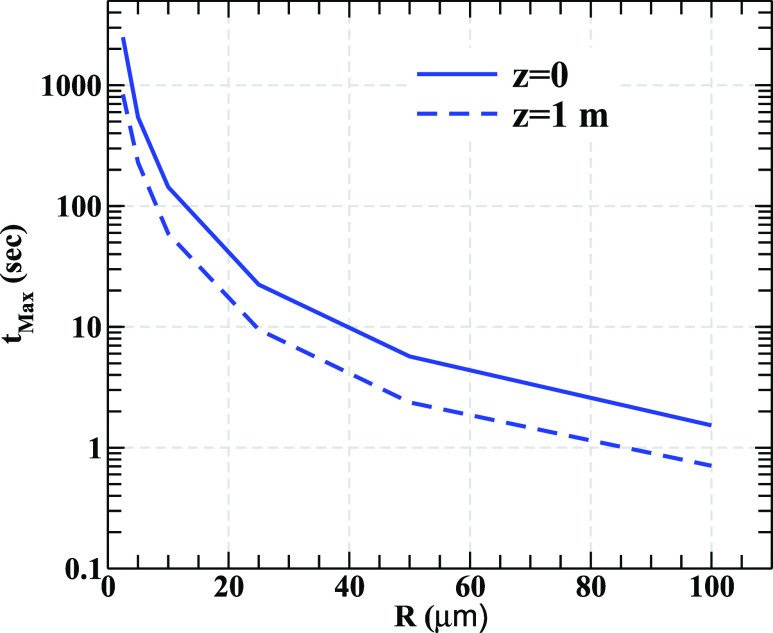
The variation in the maximum time the droplets take to reach a height of 1 m from the ground (dashed line) and to hit the ground (solid line).

How long a droplet takes to gravitationally settle on the ground or in other words how long it remains suspended in air after it is ejected through sneezing or coughing? In Fig. 4, the maximum time (*t*_max_) of suspension of the droplet in air is plotted as a function of *R* for *V*_0_ = 21 m/s for *T* = 30 °C. We find that the dependence of *t*_max_ on *V*_0_ is mild. The results clearly indicate that *t*_max_ decreases with the increase in *R*, i.e., the smaller droplets remain suspended in air for a longer time. We find that a droplet of size 100 *μ*m floats in air only for 1.5 s approximately. For large (massive) droplets, the gravitational force dominates over drag and diffusion and, consequently, they settle on the ground quickly. However, a droplet of smaller size (hence, lighter too) of 2.5 *μ*m radius survives in air for about 41 min (Fig. 4) for *u*_0_ = 0.1 m/s because for such lighter droplets, the effect of gravity is small. This result may be used as a guideline to determine the temporal distance that a healthy individual should maintain from an infected one. A healthy individual should not only be careful in maintaining the geometric distance from an infected individual but also deter the suspended lighter droplets by suitably covering nose, mouth, etc., by using a mask^12,30–32^ and other possible accessories. Moreover, the majority of droplets ejected from the exhalation process have a radius of around 10 *μ*m (see Ref. 27 and references therein) for which the use of a mask is necessary. Figure 4 also displays the maximum time taken by the droplets of various sizes to fall at a height of 1 m from its released position at a height of 1.7 m from the ground.

## IV. DISCUSSIONS

The maximum time of suspension in air and the maximum horizontal distance traveled by the droplets ejected by infected individuals through coughing and sneezing have been estimated both for still and flowing air conditions by solving the Langevin equation. All the possible forces (drag, diffusive, and gravitational) which influence the dynamics of the droplets in air have been taken into account under varying conditions of temperature, humidity, and air flow. The sizes and the initial ejection velocities used in the calculations have been taken from measured values available in the literature.^26,27^ With all these inputs, the Langevin equation has been solved rigorously to find that the small droplets travel a larger distance and remain suspended in air for a longer time under the influence of air flow, making the use of a mask mandatory to prevent the virus. Therefore, the maintenance of only stationary separation between healthy and infected individual is not substantiated. Calculations based on fluid dynamics^33^ show that small droplets have the ability to carry the pathogens a longer distance, which corroborates the fact that the maintenance of only six feet social distancing is not sufficient to evade the virus.

We have studied the impact of diffusion, represented by the term *ξ*(*t*) appearing in the right-hand side of Eq. (2), on droplets of different sizes. It is found that droplets with ≤5 *μ*m radii are affected considerably. For example, the time of suspension of a droplet of 2.5 *μ*m radius in air is changed by about 25% as a result of diffusion. It is found that smaller droplets follow the zig-zag paths with slight variations around its trajectory and stay longer in air due to diffusion. However, the impact of diffusion on larger droplets is insignificant.

We notice that such a result goes along with the very recent finding^34^ in two Wuhan hospitals, that the micrometer and sub-micrometer droplets of SARS-COV-2 were found at a distance of about 3 m from the infected patient’s bed to the room’s corners, where, indeed, the air flow is damped and/or twirls into local vortices.

It may be mentioned here that smaller droplets may originate from the fragmentation^35^ or evaporation^36^ of the larger droplets and remain suspended in air for a longer time causing potential health problems. Again in such cases, preservation of static separation is not justifiable. The isolated virus may be created from the process of evaporation. The survivability of these viruses in air for more than an hour has been reported.^37^ Such virus will remain suspended in air for a longer time due to the dominant actions of drag and diffusive forces as well as air flow as the gravitational influence on them is weak. However, it is important to mention here that in a very interesting recent work,^38^ it has been pointed out that by the processes of sneezing and coughing not only droplets are produced, but also multi-phase turbulent gas cloud, which can carry cluster of droplets of all possible sizes. In such a scenario, these contained droplets can avoid evaporation and, hence, can live longer than isolated droplets.

We have performed a thorough study considering possible effects coming from the higher order correction, associated with large Reynolds number, with respect to the Stokes approximation, but we have found only negligible changes for small droplets (*R* ≤ 10 *μ*m) that can be discarded at the level of accuracy relevant in this context (for large droplets, the action of gravity dominates over viscous force). We have also studied the impact of the temperature on the space–time evolution of the trajectories exploring a wide range from 0 °C to 40 °C, and the results show a limited impact, that is, of about 10%.

## DATA AVAILABILITY

The data that support the findings of this study are available within the article.
